# Therapeutic applications and challenges in myostatin inhibition for enhanced skeletal muscle mass and functions

**DOI:** 10.1007/s11010-024-05120-y

**Published:** 2024-09-28

**Authors:** Brock Wetzlich, Benard B. Nyakundi, Jinzeng Yang

**Affiliations:** Department of Human Nutrition, Food and Animal Sciences, University of Hawaii at Manoa, Honolulu, HI 96822 USA

**Keywords:** Myostatin, Myostatin inhibitors, Skeletal muscle, Muscular dystrophy, Obesity, Diabetes, Orthopedic disease, Clinical trials

## Abstract

Myostatin, a potent negative regulator of skeletal muscle mass, has garnered significant attention as a therapeutic target for muscle dystrophies. Despite extensive research and promising preclinical results, clinical trials targeting myostatin inhibition in muscle dystrophies have failed to yield substantial improvements in muscle function or fitness in patients. This review details the mechanisms behind myostatin’s function and the various inhibitors that have been tested preclinically and clinically. It also examines the challenges encountered in clinical translation, including issues with drug specificity, differences in serum myostatin concentrations between animal models and humans, and the necessity of neural input for functional improvements. Additionally, we explore promising avenues of research beyond muscle dystrophies, particularly in the treatment of metabolic syndromes and orthopedic disorders. Insights from these alternative applications suggest that myostatin inhibition may hold the potential for addressing a broader range of pathologies, providing new directions for therapeutic development.

## Introduction

Myostatin (MSTN), also known as growth and differentiation factor 8 (GFD8), is a member of the transforming growth factor β (TGF-β) superfamily of signaling proteins, and functions as a negative regulator of skeletal muscle mass. It was first described by McPherron et al. in 1997, where MSTN-knockout mice displayed a super-muscled phenotype. MSTN is highly conserved among mammalian species, and natural mutations have been observed to cause increased muscle mass in cattle [1–3], dogs [4], sheep [5], and humans [6]. Conversely, overexpression of MSTN causes muscle atrophy [7].

MSTN’s relationship with muscle growth has led to the widespread study of its inhibition for the treatment of muscle, bone, and metabolic diseases, as well as enhancing agricultural meat production [8–11]. Clinical trials utilizing MSTN inhibitors began in the early 2000s, primarily aiming to increase muscular function and survivability in muscular dystrophies. Despite achieving widespread success in preliminary animal trials, the journey to market for MSTN-based drugs has been largely disappointing, as none of the clinically tested inhibitors have been approved for mediating muscle mass [12–14]. Although the clinical failures of muscular dystrophy-targeting drugs have branded MSTN inhibitors as a fruitless endeavor, recent advancements in inhibitor application and design offer promising prospects for developing viable MSTN therapeutics. The purpose of this review is to provide an updated overview of current research on MSTN inhibition in the treatment of various pathologies. Specifically, we seek to provide a better understanding of clinical developments and investigate the underlying reasons for the high rate of trial failures. Lastly, we explore potential inhibitor design choices and understudied pathologies that may be better suited for MSTN-inhibition therapeutics.

## Mechanism of myostatin actions

**Fig. 1:**
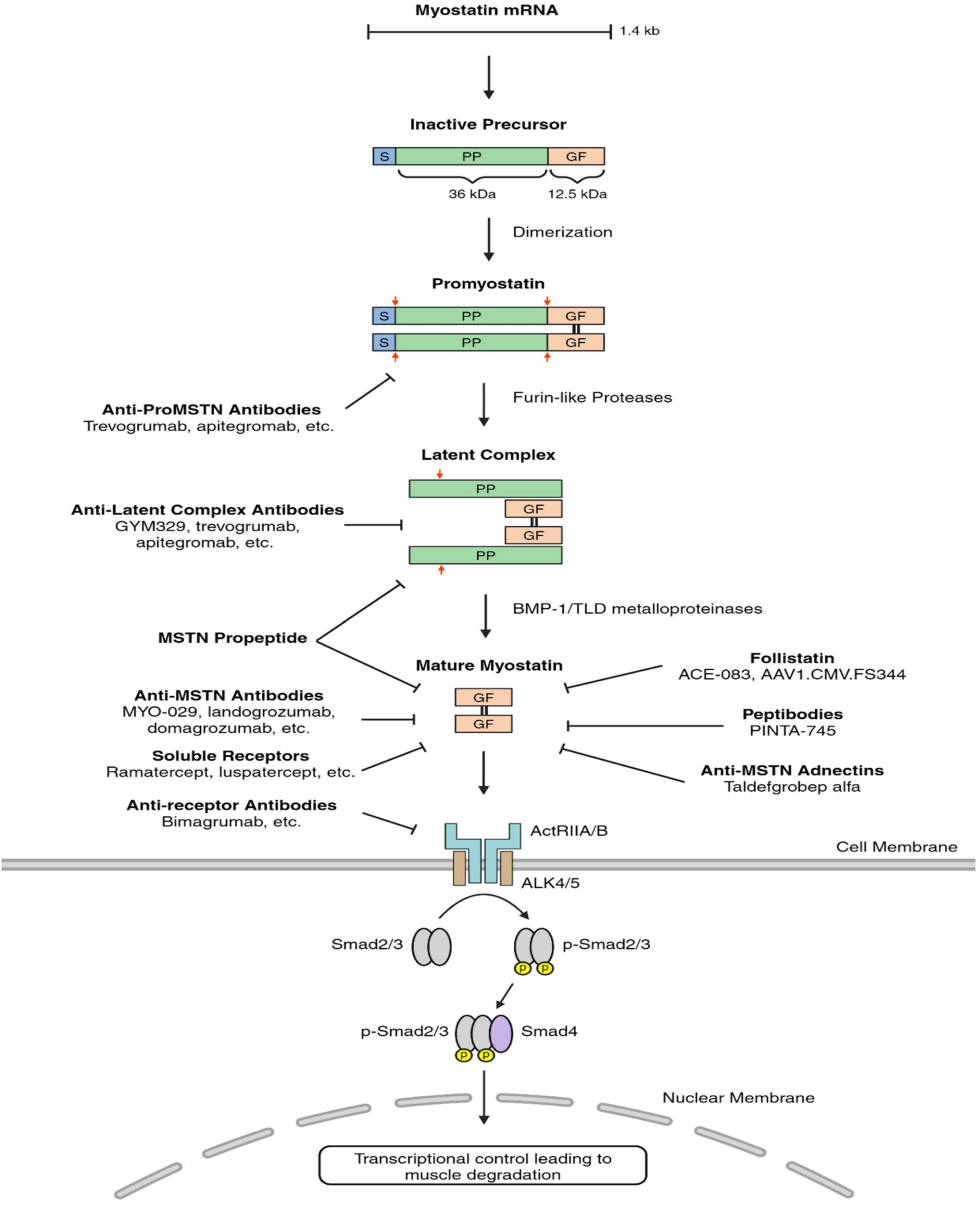
Myostatin Activation and SMAD Signaling Pathway, Including Inhibitor Targets. MSTN is secreted as an inactive precursor composed of a signal peptide (S), a 36 kDa propeptide region (PP), and a 12.5 kDa mature, or growth factor (GF) region. In the endoplasmic reticulum, GF regions dimerize, forming promyostatin. Proteolytic processing occurs as detailed in section “Mechanism of myostatin actions”. ProMSTN is cleaved at a dibasic RX site between the S and PP regions, followed by furin-like protease cleavage at an RXXR site between the PP and GF regions, indicated by red arrows. This results in a latent complex of dimerized GF and PP regions held together via non-covalent interactions. MSTN activation occurs through the cleavage of an arginine residue in the supporting PP regions, releasing the mature MSTN dimer. Mature MSTN binds to ActRIIB or ActRIIA, initiating receptor dimerization and recruitment of ALK4/5, triggering various signaling pathways described in section “Mechanism of myostatin actions”. The SMAD pathway is depicted, where phosphorylated Smad2/3 forms a complex with Smad4 and translocates into the nucleus, regulating gene expression associated with muscle homeostasis. The figure also highlights the targets of various MSTN inhibitors discussed in section “Inhibition of Myostatin in animal and human studies”

MSTN is primarily expressed in skeletal muscle but is also expressed to a lesser extent in adipose tissue [15], heart [16], and kidney [17]. Like most other members of the TGF-β family, MSTN is secreted as an inactive precursor, comprised of an N-terminal signal peptide, N-terminal propeptide, and C-terminal growth factor (GF) domain [18]. In the endoplasmic reticulum, the MSTN precursor undergoes dimerization at the C-terminus, forming a complex known as promyostatin (proMSTN). Initially, the signal peptide is removed by signal peptidase. Furin-like proteases then cleave the MSTN precursor at a conserved tetrabasic site of RXXR amino acid sequence located between the propeptide and GF regions, resulting in an inactive complex consisting of dimerized GF peptides with supporting propeptide regions, which are non-covalently bound to keep MSTN in a latent state (Fig. 1) [19–21]. This pro-form of MSTN has higher abundance and longevity than its active mature form, which has a shorter temporal and spatial activity window [20, 22]. MSTN is activated following the cleavage of its propeptides by bone morphogenetic protein-1 (BMP-1)/tolloid (TLD)-like metalloproteinases at an arginine residue. This action releases the propeptides from the dimerized GF region, allowing mature MSTN to interact with non-specific activin receptors (ActRII) on the surface of target cells (i.e., myoblasts) through a paracrine pathway [23, 24]. The MSTN pathway is classically initiated through MSTN binding to ActRIIA/B receptors, with a notably higher affinity for ActRIIB. This binding induces dimerization, which subsequently activates activin-like kinase (ALK4 or ALK5). This receptor activation ultimately leads to the phosphorylation of SMAD2 and SMAD3, which then form a complex with SMAD4. The SMAD complex translocates into the nucleus and negatively regulates myoblast cell activities by modulating gene expressions (Fig. 1). This, in turn, results in the expression of various atrophic E3-Ubiquitin ligases such as Atrogin1 and muscle RING-finger protein-1 (MuRF1) [23, 25, 26]. Additionally, MSTN plays a role in signaling the mitogen-activated protein kinase (MAPK) pathway, specifically the c-Jun N-terminal kinase (JNK), p38, and extracellular signal-regulated kinases (ERK) pathways. These pathways are known to inhibit the transcriptions of a variety of myogenesis-related genes [27–29].

MSTN plays a pivotal role in skeletal muscle development. During fetal development, muscle fiber formation occurs, and MSTN mRNA is highly expressed in the developing skeletal muscle. In mice, expression begins around 9.5 days post-coitum (dpc) and peaks at approximately 14.5 dpc, indicating its critical role in regulating early muscle growth [30]. Following birth, MSTN levels remain high, though not as elevated as during fetal development [30]. As adulthood sets in, MSTN levels stabilize, maintaining muscle homeostasis [30]. MSTN-knockout animals, lacking MSTN presence during prenatal or neonatal development, exhibit a dramatic 2- to 3-fold increase in muscle mass compared to wild-type animals [30]. Homozygous mutant mice display approximately 30% more body weight, with both larger muscles fibers in cross-sectional area (hypertrophy) and a greater in fiber number (hyperplasia). Furthermore, MSTN-knockout animals demonstrate a higher proportion of type II fibers and a reduced number of type I fibers, along with decreased adipose tissue [30–32]. Postnatal suppression of MSTN, achieved through conditional gene targeting or the administration of MSTN inhibitors such as its propeptide, antibody, or follistatin, induces significant but relatively lesser increases in skeletal muscle mass [33–35]. In contrast to MSTN-knockout models, muscle growth from postnatal suppression of MSTN results solely from muscle hypertrophy, not hyperplasia, but still predominantly induces type II muscle fibers [36–38].

## Inhibition of myostatin in animal and human studies

Since its initial description by McPherron et al. in 1997, MSTN inhibition has been widely considered as a potential treatment for muscle wasting diseases. Indeed, the focus of most clinical studies to date has been on diseases such as Duchenne muscular dystrophy (DMD), sporadic inclusion body myositis (sIBM), and limb-girdle muscular dystrophy (LGMD). Muscular dystrophies are genetic disorders characterized by muscle weakness and degeneration resulting from mutations in specific genes. These conditions often lead to mobility issues and premature death [39]. While MSTN inhibition does not address the underlying gene mutations responsible for these diseases, it is anticipated to offer a universal approach to treating muscle wasting that does not rely on correcting individual molecular defects, which can vary between patients and types of muscular dystrophy. Other conditions characterized by muscular wasting, such as sarcopenia and cancer cachexia, are also expected to benefit from improvements in muscular function. Preclinical studies conducted in MSTN-null mdx mice, a model of DMD and Becker muscular dystrophy (BMD) featuring a premature stop codon in the gene for dystrophin, have demonstrated increased muscle size and strength [40]. Furthermore, a three-month treatment regimen with anti-MSTN antibodies in mdx mice resulted in enhancements in body weight, muscle mass, muscle size, and absolute muscle strength, accompanied by a significant reduction in muscle degeneration [41]. Given the consistent success in ameliorating muscle wasting diseases in animal models, various inhibitors have been developed for clinical evaluation (Table 1).

**Table 1:** Overview of clinical trials of myostatin inhibitors as per www.clinicaltrials.gov (Access date August 8, 2024)

Name(s)	Sponsor	Type	Target	First Trial	Remarks	Clinical trials [NCT# (Phase, Conditions, Completion Date)]
MYO-029 (Stamulumab)	Wyeth	Antibody	Mature MSTN	2004–10	Discontinued due to not meeting endpoints in improvements to muscular strength/function (Wagner et al., 2008)	NCT00563810 (Phase 1, Healthy, 2006,04), NCT00104078 (Phase 1/2, BMD/FMD/LGMD, 2007–01)
Landogrozumab (LY-2495655)	Eli Lilly	Antibody	Mature MSTN	2008–01	Discontinued for not meeting endpoints in muscle strength/function and survivability (Woodhouse et al., 2016; Golan et al., 2018)	NCT01524224 (Phase 1, Cachexia, 2016–01), NCT01341470 (Phase 1, Healthy, 2015–05), NCT01369511 (Phase 2, Atrophy, 2014–02), NCT01505530 (Phase 2, Cachexia, 2016–01), NCT01604408 (Phase 2, Muscle weakness, 2013–12)
Sotatercept (ACE-011, MK-7962)	Merck/Acceleron	ActRIIA Fusion Protein	Mature MSTN / Various	2008–01	Designed to target various TGF-β proteins, not designed to target MSTN. Results on muscle mass/function not reported. Studies ongoing. Superseded by luspatercept	NCT05818137 (Phase 3, PAH), + 22 more (MPN, Anemia, Beta-thalassemia, Est. 2025–08)
Ramatercept (ACE-031/ RAP-031/ HGT-4510)	Acceleron	ActRIIB Fusion Protein	Mature MSTN	2008–09	Terminated due to non-muscle-related side effects (Campbell et al., 2017)	NCT00755638 (Phase 1, Healthy, 2009–07), NCT00952887 (Phase 1, Healthy, 2011–02), NCT01099761 (Phase 2, DMD, 2011–06), NCT01239758 (Phase 2, DMD, 2011–05)
PINTA-745 (AMG-745)	Amgen/Atara Biotherapeutics	Peptibody Fusion Protein	Mature MSTN	2010–04	Discontinued due to not meeting endpoints in improvements to muscular strength/function (Padhi et al., 2014)	NCT00975104 (Phase 2, Sarcopenia, 2011–08), NCT01958970 (Phase 1/2, End stage renal disease, kidney disease, protein energy wasting, 2016–01)
Bimagrumab (BYM-338)	Lily, Novartis	Antibody	ActRIIA/ActRIIB	2011–08	Studies are ongoing for obesity	NCT01423110 (Phase 2, sIBM, 2012–05), NCT01433263 (Phase 2, Cachexia, 2014–04), NCT01601600 (Phase 2, Sarcopenia, 2013–12), NCT01669174 (Phase 2, COPD + Cachexia, 2014–12), NCT01925209 (Phase 2/3, sIBM, 2016–01), NCT02250443 (Phase 2/3, sIBM, 2016–08), NCT01868685 (Phase 2, Prolonged mechanical ventilation, 2015–01), NCT02152761 (Phase 2, Atrophy, 2018–10), NCT02333331 (Phase 2, Sarcopenia, 2018–06), NCT02468674 (Phase 2, Sarcopenia, 2018–12), NCT02573467 (Phase 3 sIBM, 2017–02), NCT03005288 (Phase 2, T2DM, 2019–05), NCT05616013 (Phase 2, Obesity, Est. 2025–06), NCT05933499 (Phase 2, Obesity, Est. 2028–08)
Luspatercept (ACE-536, REBLOZYL®)	Merck/Acceleron	ActrIIB Fusion Protein	Mature MSTN / Various	2011–09	Designed to target various TGF-β proteins, not designed to target MSTN. Results on muscle mass/function not reported. FDA approved for MDS anemia	NCT04064060 (Phase 3, MPN, MDS, Beta-thalassemia, Est.2028–05), + 40 more (Anemia, Anemia-related)
Trevogrumab (REGN1033, SAR391786)	Regeneron	Antibody	proMSTN	2012–01	Studies ongoing. Recruiting for obesity trial	NCT01507402 (Phase 1, Healthy, 2012–11), NCT01720576 (Phase 1, Healthy, 2013–06), NCT01910220 (Phase 1, Healthy, 2014–10), NCT01963598 (Phase 2, Sarcopenia, 2015–02), NCT02741739 (Phase 1, Healthy, 2016–05), NCT02943239 (Phase 1, Healthy, 2019–04), NCT03710941 (Phase 2, sIBM, 2020–11), NCT06299098 (Phase 2, Obesity, Est. 2026–06)
Domagrozumab (PF-06252616)	Pfizer	Antibody	Mature MSTN	2012–06	Discontinued due to not meeting endpoints in improvements to muscular strength/function	NCT01616277 (Phase 1, Healthy, 2014–08), NCT02310763 (Phase 2, DMD, 2018–11), NCT02841267 (Phase 1b/2, LGMD, 2019–01), NCT02907619 (Phase 2, DMD, 2018–11)
AAV1.CMV.FS344(rAAV1.CMV huFollistatin344)	Nationwide Children's Hospital/Milo Biotechnology/Jerry R. Mendell	Follistatin AAV	Mature MSTN	2012	No studies currently active	NCT01519349 (Phase 1, BMD, sIBM, 2017–10), NCT02354781 (Phase 1/2, DMD, 2017–11)
Taldefgrobep alfa (BHV2000, RO7239361, BMS-986089, RG6206, Talditercept alpha)	Biohaven Pharmaceuticals/Roche/Bristol Myers	Adnectin Fusion Protein	Mature MSTN	2014–06	Research in DMD discontinued due to failure to meet endpoints. Studies ongoing for treatment of SMA	NCT02145234 (Phase 1, Healthy, 2016–02), NCT03100630 (Phase 1, Healthy, 2017–10), NCT02515669 (Phase 1/2, DMD, 2020–04), NCT03039686 (Phase 2/3, DMD, 2020–04), NCT05337553 (Phase 3, SMA, Est. 2025–01)
ACE-083	Acceleron	Follistatin Fusion Protein	Mature MSTN	2014–09	Discontinued due to not meeting endpoints in improvements to muscular strength/function	NCT02257489 (Phase 1, Healthy, 2016–04), NCT02927080 (Phase 2, FSHD, 2019–10), NCT03124459 (Phase 2, CMT, 2020–03), NCT03943290 (Phase 2, CMT/FSHD, 2020–03)
ACE-2494	Acceleron	Ligand Trap	Mature MSTN	2018–02	Discontinued due to high frequency of anti-drug antibodies (https://www.businesswire.com/news/home/20190404005769/en/Acceleron-Discontinues-Development-of-Phase-1-Molecule-ACE-2494)	NCT03478319 (Phase 1, Healthy, 2019–06)
Apitegromab (SRK-015)	Scholar Rock	Antibody	proMSTN	2019–04	Ongoing, all studies currently active	NCT03921528 (Phase 2, SMA, Neuromuscular Diseases,2024–02), NCT05156320 (Phase 3, SMA, Neuromuscular Diseases, Est. 2024–12), NCT05626855 (Phase 3, SMA, Neuromuscular Diseases, Est. 2027–01) NCT06445075 (Phase 2, Obesity, Est. 2025–10)
BLS-M22	BioLeaders Corporation	L. casei vaccine	Mature MSTN	2019–06	Study completed; no results posted	NCT03789734 (Phase 1, Healthy, 2020–11)
GYM329 (RO7204239)	Roche	Antibody	proMSTN	2022–06	Combination treatment with Risdiplam. Ongoing, all studies currently recruiting	NCT05115110 (Phase 2/3, SMA, Est. 2026–06), NCT05548556 (Phase 2, FSHD, Est. 2026–10)
Follistatin Plasmid	Minicircle	Follistatin-Producing Plasmid	Mature MSTN	2022–08	No results posted	NCT06411366 (Phase 1, Healthy/Aging, 2023–08)

*AAV* adeno-associated virus, *BMD* Becker muscular dystrophy, *CMT* Charcot-Marie-Tooth disease, *COPD* chronic obstructive pulmonary disease, *DMD* Duchenne muscular dystrophy, *FMD* facioscapulohumeral muscular dystrophy, *FSHD* facioscapulohumeral muscular dystrophy, *LGMD* limb-girdle muscular dystrophy, *MDS* myelodysplastic syndromes, *MPN* myeloproliferative neoplasms, *MSTN* myostatin, *PAH* pulmonary hypertension, *sIBM* sporadic inclusion body myositis, *SMA* spinal muscular atrophy, *T2DM* type 2 diabetes mellitus

### Antibodies

The majority of clinically tested MSTN inhibitors have been MSTN-based antibodies. In 2004, Wyeth Pharmaceuticals (now owned by Pfizer) developed the monoclonal anti-MSTN antibody MYO-029, the first MSTN inhibitor to enter clinical trials. MYO-029 binds to mature MSTN, thereby preventing its interaction with ActRIIA/B receptors. Despite conducting Phase 1 and Phase 2 trials involving patients with BMD, facioscapulohumeral muscular dystrophy (FSHD), and LGMD, administering 2 doses of 1–10 mg/kg MYO-029 every 2 weeks for 6 months found no significant improvements in muscle size, strength, or function, leading to the discontinuation of further development [14].

After MYO-029, the next MSTN inhibitor to reach clinical trials was landogrozumab, an anti-MSTN monoclonal antibody developed by Eli Lilly. Landogrozumab was tested in phase 2 trials targeting sarcopenia, sarcopenia related to hip surgery, and cachexia. Following 5 equally spaced treatments of 315 mg over 20 weeks in patients aged 75 years or older, those who received landogrozumab showed an increase of 0.44 kg in appendicular lean body mass. Additionally, they demonstrated significant improvements in stair climbing (four-step: −0.46 s, 12-step: −1.28 s), chair rising with arms (− 4.15 s), and fast gait speeds (+ 0.05 m/s) [42]. However, subsequent trials failed to show significant increases in appendicular lean mass among patients with osteoarthritis scheduled for elective total hip arthroplasty [43]. Similarly, no improvements in muscle function or survival were observed in pancreatic cancer patients with cachexia [44]. Nonetheless, the former of the two trials did reveal a statistically significant, yet relatively low increase in lean muscle mass [43].

Domagrozumab, developed by Pfizer, is another anti-MSTN/GDF-11 monoclonal antibody, similar in design to its predecessors MYO-029 and landogrozumab. In studies on mdx mice, domagrozumab significantly boosted body weight, muscle weight, and grip strength [45], demonstrating greater enhancements in muscular strength compared to MYO-029 [46]. However, a series of phase 1 and 2 clinical trials targeting DMD and LGMD did not meet their endpoints, with no significant improvement in muscle strength, function, or size among subjects [47, 48]. Further development of domagrozumab was ultimately terminated in 2018 due to the lack of positive results from these studies [49].

Bimagrumab, originally developed by Novartis and later acquired by Lilly, is an antibody targeting MSTN/Activin receptor ActRIIB and ActRIIA, thereby preventing MSTN from binding to the receptor. Initial trials of bimagrumab showed promise, with a 5.7% increase in lean muscle mass and a 14.6% improvement in 6-min walking distance (6MWD) in patients with sIBM given a single dose of 30 mg/kg [50]. However, more extensive studies on sIBM found no improvements in 6MWD and overall mobility [51, 52]. Subsequent studies produced mixed results: administering a single or twice doses of 30 mg/kg bimagrumab to sarcopenia-affected patients resulted in an approximately 6.5% increase in thigh muscle volume (TMV). Notably, improvements in gait speed and 6MWD were explicitly observed in slower walkers, with a mean increase of 0.15 m/s in gait speed and 82 m in 6MWD at Week 16 compared to those on placebo [53]. However, no changes were observed when 700 mg bimagrumab was given monthly to patients following an optimized standard of care for diet and exercise [54]. Additionally, two doses of 30 mg/kg bimagrumab in patients with chronic obstructive pulmonary disease led to an approximate 5% increase in TMV after 24 weeks but did not improve functional capacity [55]. Similarly, bimagrumab administered every 4 weeks for 24 weeks to older adults recovering from hip fracture showed a significant dose-dependent increase in muscle mass up to a mean change of 2.8 kg, but no functional benefit [56]. Clinical trials for bimagrumab are ongoing.

In addition to mature MSTN-targeting antibodies, various other designs exist that target different stages of MSTN expression. One notable example is trevogrumab, developed by Regeneron. It is a monoclonal antibody with an IgG4 Fc domain designed for sarcopenia and inclusion body myositis (IBM) treatment. Trevogrumab targets MSTN in its mature, latent, and pro-forms without cross-reactive binding to GDF11 [57]. Apitegromab, developed by Scholar Rock, specifically targets MSTN in its latent form by stabilizing its conformation, thereby preventing access to prodomain protease cleavage sites [58, 59]. Apitegromab has shown efficacy in increasing muscle mass and function in mouse models of spinal muscular atrophy (SMA) [60]. Similarly, GYM-329 by Roche is designed to treat FSHD by binding to latent MSTN, thus blocking its conversion to its mature form [61]. These antibodies are currently undergoing clinical trials or awaiting the reporting of results.

### Fusion proteins

Another class of MSTN inhibitors is fusion proteins, often in the form of soluble activin receptors, which act as ligand traps by binding MSTN and preventing its further interactions. Acceleron, now owned by Merck, first brought ligand traps to clinical trials with ramatercept in 2008. Ramatercept is a fusion protein consisting of human IgG linked to the extracellular domain of ActRIIB, acting as a soluble form of ActRIIB, which binds MSTN and other TGF-β members. Ramatercept showed promise in both phase 1 and phase 2 trials. In a phase 1 trial involving women, total muscle volume (TMV) was improved by approximately 5.1% following a single injection of 3 mg/kg [62]. Additionally, in a phase 2 trial conducted with boys affected by DMD, significant improvements were observed in muscle mass, with the group treated with 1 mg/kg every 2 weeks experiencing an approximate mean increase of 4–5%. Moreover, improvements were noted in 6-min walk distance (6MWD), bone mineral density, and reductions in fat mass [12, 63]. However, non-muscle-related adverse side effects including nosebleeds, gum bleeding, telangiectasia, and erythema led to the discontinuation of further study [12].

Acceleron’s luspatercept is akin to its predecessor, ramatercept, but contains modifications in the extracellular domain of ActRIIB to promote binding of various TGF-β proteins. Luspatercept began clinical trials in 2011 for the treatment of hematologic disorders and demonstrated efficacy in increasing hemoglobin counts in patients with anemia [64, 65], potentially attributed to a reduction in SMAD signaling leading to enhanced erythroid maturation [66]. Luspatercept received FDA approval in 2020 for the treatment of anemia associated with myelodysplastic syndromes, myelofibrosis, and beta-thalassemia [67]. However, the effect of luspatercept on MSTN expression and muscle modulation has not been reported.

In contrast to receptor-based ligand trap designs like ramatercept and luspatercept, PINTA-745, initially developed by biotech company Amgen and later by Atara Biotherapeutics, is a distinctive peptibody fusion protein comprised of a human IgG FC with a MSTN-neutralizing peptide. In a post-stroke muscle loss mouse model, PINTA-745 demonstrated a significant increase in muscle mass, strength, and motor function [68]. However, in a phase 1/2 trial involving patients with prostate cancer undergoing androgen deprivation therapy, weekly injections of 3 mg/kg PINTA-745 for four weeks resulted in a modest 2.2% increase in muscle size, but showed no improvement in physical strength assessed through the Short Physical Performance Battery or the one-repetition maximum knee extension test [13]. These outcomes led to the discontinuation of further development.

Taldefgrobep alfa, developed by Bristol-Meyers-Squibb and later by Biohaven Pharmaceuticals, is a unique anti-MSTN adnectin. It utilizes an engineered scaffold based on the 10th fibronectin type III domain coupled with a human IgG Fc domain, exhibiting a binding affinity for the C-terminal of mature MSTN and the ActRIIB–MSTN complex, similar to an antibody [69]. Taldefgrobep binding prevents ALK-4/5 recruitment, thereby inhibiting the SMAD pathway. In a phase 1 trial involving healthy adults, a 24-week treatment period with weekly doses ranging from 45 to 150 mg led to a universal decrease in free MSTN by ≥ 90%. Additionally, significant increases in lean body mass, up to a mean increase of 2.69%, and thigh muscle volume, up to a mean increase of 4.75%, were observed [69]. In phase 2 trials with boys affected by DMD, weekly doses of taldefgrobep alfa led to a modest 4.9% increase in lean body mass index in the pooled treatment group compared to placebo, but ultimately found no change in motor function [69]. Further research targeted toward DMD has been terminated, but a phase 3 study evaluating taldefgrobep alfa in SMA is currently underway.

### Follistatin

Follistatin (FST) and its related FST-type molecules are naturally antagonists to several TGF-β proteins, and are widely known inhibitors of MSTN [70, 71]. FST binds to mature MSTN with high affinity and inhibits its binding to ActRIIB, but does not interact with proMSTN [20, 72]. The N-terminal α-helical domain of FST interacts directly with a type I receptor binding site of MSTN, causing inactivation [73]. Consequently, FST lacking its C-terminal peptide or fragments of the N-terminal region show similar inhibitory effects [73–75]. Transgenic overexpression of FST results in a 2- to 3-fold increase in muscle mass via hypertrophy and hyperplasia [21]. However, the increase in muscle mass is not solely attributed to blocking MSTN, as FST also inhibits the activities of multiple TGF-β family members, some of them play a role in limiting muscle mass [76]. FST-overexpressing MSTN-null mice display an even more extreme fourfold increase in muscle [77]. Contrarily, FST-null mice have reduced muscle mass at birth and perish within a few hours [78]. Delivery of FST-coding mRNA gene therapy and AAV, as well as follistatin peptide derivatives, have all been shown to produce substantial muscle increase in animal models [35, 79, 80].

The number of clinically tested follistatin-based methods is limited. ACE-083, developed by Acceleron/Merck, is a fusion protein consisting of a human IgG2 Fc domain linked with a modified human FST. ACE-083 is designed for intra-muscular injection and causes localized MSTN inhibition. Preclinical studies of ACE-083 demonstrated a dramatic increase in muscle mass and strength in wild-type, Charcot-Marie-Tooth disease (CMT), and DMD disease model mice [81]. In a phase 1 study, ACE-083 injected in to the rectus femoris muscle of healthy women resulted in approximately a 14.5% increase in local muscle mass but no change in muscle strength [82]. Phase 2 studies in patients with FSHD or CMT also revealed significant localized increases in muscle mass but failed to demonstrate any improvement in muscle function [83, 84]. The failure of ACE-083 to meet clinical endpoints ultimately led Acceleron to discontinue its development and shift its focus of TGF-β targeting therapeutics away from muscle-dystrophic diseases.

AAV1.CMV.FS344, developed by Milo Biotechnology/Nationwide Children's Hospital, is a modified human follistatin isoform delivered by adeno-associated virus (AAV) to suppress MSTN. This follistatin AAV has shown promising results in mice, with a single injection significantly increasing long-term muscle mass and strength in wild-type, aged, and mdx models [85]. In humans, AAV.CMV.FS344 has undergone limited testing in a few stage 1 and 2 clinical trials. Administration of the AAV to BMD patients showed improvements to 6MWT, although there was no control group, and only six subjects were tested [86]. AAV injection in a similarly small group of sIBM patients also resulted in increased 6MWT performance [87]. Study of AAV.CMV.FS344 has yet to be terminated, but the continued lack of participants in each study raises questions about the validity of the results.

### MSTN propeptide

The MSTN propeptide is a potent inhibitor of MSTN but has seemingly been overlooked in clinical research. MSTN propeptide functions as an inhibitor by binding to the mature MSTN dimer, reverting it to its latent state [88]. Furthermore, two propeptides can bind to the dimerized proMSTN N-terminal region via a critical peptide sequence, preventing the proteolytic processing of proMSTN into its mature form [18]. Transgenic mice overexpressing MSTN propeptide exhibit a dramatic muscling phenotype, with a 17–30% increase in body weight [37]. The inhibitory binding capabilities of MSTN propeptide are due to specific amino acid residues in the propeptide's N-terminus (42–115AA) [18]. Smaller minimum inhibitory peptide sequences (< 25AA) of MSTN propeptide have been identified and proven to inhibit MSTN in vivo [89]. Mutations of MSTN propeptide at the arginine-residue cleavage site can enhance the effectiveness of MSTN inhibition by rendering the propeptide resistant to cleavage by BMP-1/tolloid proteinases, thereby preventing activation of the latent complex [24]. Mice injected with the mutant form of propeptide exhibited more pronounced muscling results than those given wild-type MSTN propeptide [24]. Despite its potent inhibition of MSTN in animals, no attempts have been made to utilize MSTN propeptide in clinical trials.

## Challenges in myostatin inhibition

### Specificity in inhibitor designs

Despite yielding positive results in various animal studies, MSTN inhibition has not improved human muscular function. A lack of specificity in many MSTN inhibitors could account for unsatisfactory clinical trials. MSTN shares significant structural similarities with other members of the TGF-β superfamily, particularly GDF11, showing nearly 90% sequence identity in their mature domains [90]. Consequently, many anti-MSTN antibodies inadvertently cross-react with GDF11 [91, 92], leading to cross-reaction effects or reduced efficacy. Receptor-based ligand traps encounter similar problems due to ActRIIA and ActRIIB receptors binding to GDF11, activins A, B, and AB, and BMPs 9 and 10 [93]. Inhibiting ActRIIA/B receptors will also affect the signaling of these proteins, potentially causing unintended off-target effects. Likewise, FST has also been shown to bind to GDF11, activins A, B, AB, and E, inhibins A and B, BMPs 2, 4, 6, 7, and 15 [21, 94]. Muramatsu et al. demonstrated the importance of specificity in design by utilizing GYM-329, an antibody which specifically targets the latent form of MSTN. In mice, GYM-329 was shown to increase muscle mass in 3 different models of muscle dystrophy, demonstrating a larger increase in muscle mass and grip strength compared to landogrozumab and domagrozumab, two unspecific-antibodies [61]. GYM-329 treatment also resulted in greater grip strength increases over bimagrumab, the anti-ActRIIA/B antibody [61]. Targeting the latent MSTN complex is likely more efficient not only due to specificity but also due to the increased temporal availability of the latent complex compared to the active mature dimer [20]. In addition to efficacy issues, cross-reactivity poses a serious risk of side effects. As previously mentioned, clinical studies of the soluble receptor ramatercept were prematurely halted due to significant adverse effects, such as nosebleeds, gum bleeding, telangiectasia, and erythema. These adverse events were attributed to the unintended cross-inhibition of BMP9 and BMP10, critical ligands involved in endothelial cell function [12]. Future research and development efforts for MSTN inhibitors should prioritize specificity to mitigate off-target effects and enhance efficacy.

### Availability of circulating myostatin

A difference in serum MSTN concentration between healthy and diseased individuals presents another obstacle to developing MSTN inhibitors. Most muscle atrophy and dystrophy diseases are characterized by lower concentrations of circulating myostatin [95]. Patients affected with DMD, for instance, exhibit approximately 65% lower concentrations of serum MSTN compared to healthy adults [96]. Despite a 90% reduction in MSTN compared to pre-treatment levels in DMD patients treated with domagrozumab, muscle mass did not increase significantly [96]. This suggests that the already low MSTN levels in DMD patients may reduce the effectiveness of MSTN inhibitors, as further lowering MSTN might not significantly increase muscle mass, as discussed by Mariot et al. (2017) [95]. Additionally, Mariot et al. (2017) found that in muscle wasting and atrophying diseases, not only is myostatin downregulated, but the activin receptor is also downregulated, along with an increase in the MSTN antagonist follistatin [95]. These factors further complicate the therapeutic potential of MSTN inhibitors in muscle wasting diseases.

MSTN concentrations are also affected by other conditions. Many studies generally suggest that serum MSTN is highest in young individuals and decreases with age [97, 98], which could pose challenges for using MSTN inhibitors to treat sarcopenia in older adults. Furthermore, patients suffering from cancer cachexia also show decreased MSTN concentrations compared to non-cachectic individuals [99, 100]. In patients experiencing severe muscle wasting, the decline in circulating MSTN levels may be attributed to the diminished capacity of muscles to produce myokines, including MSTN.

Much of the data about circulating MSTN levels may be questioned due to potential methodological limitations. Binding reagent assays (e.g., immuno-assays and aptamer-based methods), the most popular method for determining MSTN concentrations, have been shown to cross-react with GDF-11 [101, 102]. However, as GDF-11 is less abundant than GDF-8, its impact on overall MSTN measurements may be inconsequential [102]. Regardless, comprehensive research utilizing refined methodologies to accurately measure serum MSTN concentrations is essential to best determine if reduced MSTN impacts the effectiveness of inhibition therapies [103, 104].

The failure of MSTN inhibitors to effectively treat muscle wasting diseases in humans despite promising results in preclinical studies may stem from species-specific differences in serum MSTN levels. On average, human serum MSTN levels are around 5–10 ng/ml, whereas mice exhibit concentrations exceeding 100 ng/ml, up to a 20-fold difference [105, 106]. This disparity in MSTN availability may contribute to a shift in potency between species. A pharmacokinetic study with MYO-029 found that the concentration of MYO-029 required to elicit a 50% improvement in muscle mass in monkeys was 18 times higher compared to the same improvement in mice [107]. This difference is even more pronounced in diseased models. While mdx mice have lower serum MSTN levels (~ 50 ng/ml) than their wild-type counterparts, their concentration is much higher and proportionally less diminished compared to humans affected by DMD (~ 1 ng/ml), potentially providing a greater scope for the effects of MSTN inhibition [105]. This discrepancy in pharmacokinetics and MSTN serum concentration between diseased human and mouse models may significantly contribute to the difference in results between clinical and animal trials.

### Mediating functional strength

Even if MSTN inhibition increases muscle mass, it does not necessarily translate into improved functional strength in muscle wasting disorders. While MSTN inhibition may stimulate muscle hypertrophy, its effectiveness in improving functional strength relies heavily on synergistic motor neuron activation and mechanical signaling induced by exercise. Without adequate fusion of newly formed myotubes with existing muscle fibers, facilitated by neural input, increased muscle mass may not lead to meaningful functional improvements [108]. This limitation is especially relevant in conditions like DMD, where neuromuscular junction vulnerability and reduced neural input contribute to impaired translation of neurological signals to skeletal muscles [109]. In contrast, mdx mice typically exhibit robust contractile function and maintain ambulation throughout their lifespan, which may elucidate why MSTN inhibition in these mice can result in gains in both muscle mass and function [110]. While most clinical trials in muscular wasting disorders prioritize functional improvements as a primary endpoint, if this information holds true, the limited translation of increased muscle mass into functional strength due to neural deficits may impede the achievement of these desired functional improvements.

It is possible that the challenge of improving muscular function could be bypassed by integrating MSTN inhibition therapy with exercise. Studies in mice demonstrate that combining MSTN inhibition therapy with exercise, be it aerobic or resistance training, results in significantly enhanced muscle quality compared to either intervention alone [111, 112]. However, trials involving the combination of bimagrumab with an exercise program in sarcopenia patients, as previously mentioned, did not yield any discernible difference between groups receiving combined therapy or exercise alone [54]. Further clinical research is needed to determine if the combination of MSTN inhibition therapy with exercise could be effective in increasing muscle function in humans.

## Expanding therapeutic potential

### Myostatin inhibition in obesity, diabetes, and metabolic syndromes

While treating muscle wasting disorders has presented numerous difficulties and demonstrated limited success, inhibition of MSTN may offer a more promising approach to address other pathologies effectively. Unlike conditions like muscular dystrophy, sarcopenia, and cancer cachexia, obesity and diabetes correlate with elevated serum levels of MSTN [113]. A study surveying MSTN serum concentration in human adults found a positive correlation between obesity and increased MSTN, a positive association with insulin resistance, and a negative correlation with insulin sensitivity [114]. This cause-and-effect relationship with insulin resistance is supported by the observed increase in insulin resistance after injection of MSTN in mice [115]. Furthermore, in high-fat diet-induced obesity-susceptible C57BL/6 mice, consumption of high-fat feed led to an increase in MSTN expression, indicating that MSTN may play an essential role in mediating obesity [116]. Moreover, both type 1 and type 2 diabetes patients were shown to have higher serum MSTN concentrations when compared to healthy counterparts [117, 118]. The increased MSTN levels in obesity, insulin resistance, and diabetes suggest that MSTN-targeted inhibitors can improve metabolic function and promote weight loss in obese individuals. By blocking MSTN action, these inhibitors could enhance muscle growth, increase energy expenditure, and improve insulin sensitivity, offering a promising approach to combating diabetes-related health conditions.

**Fig. 2:**
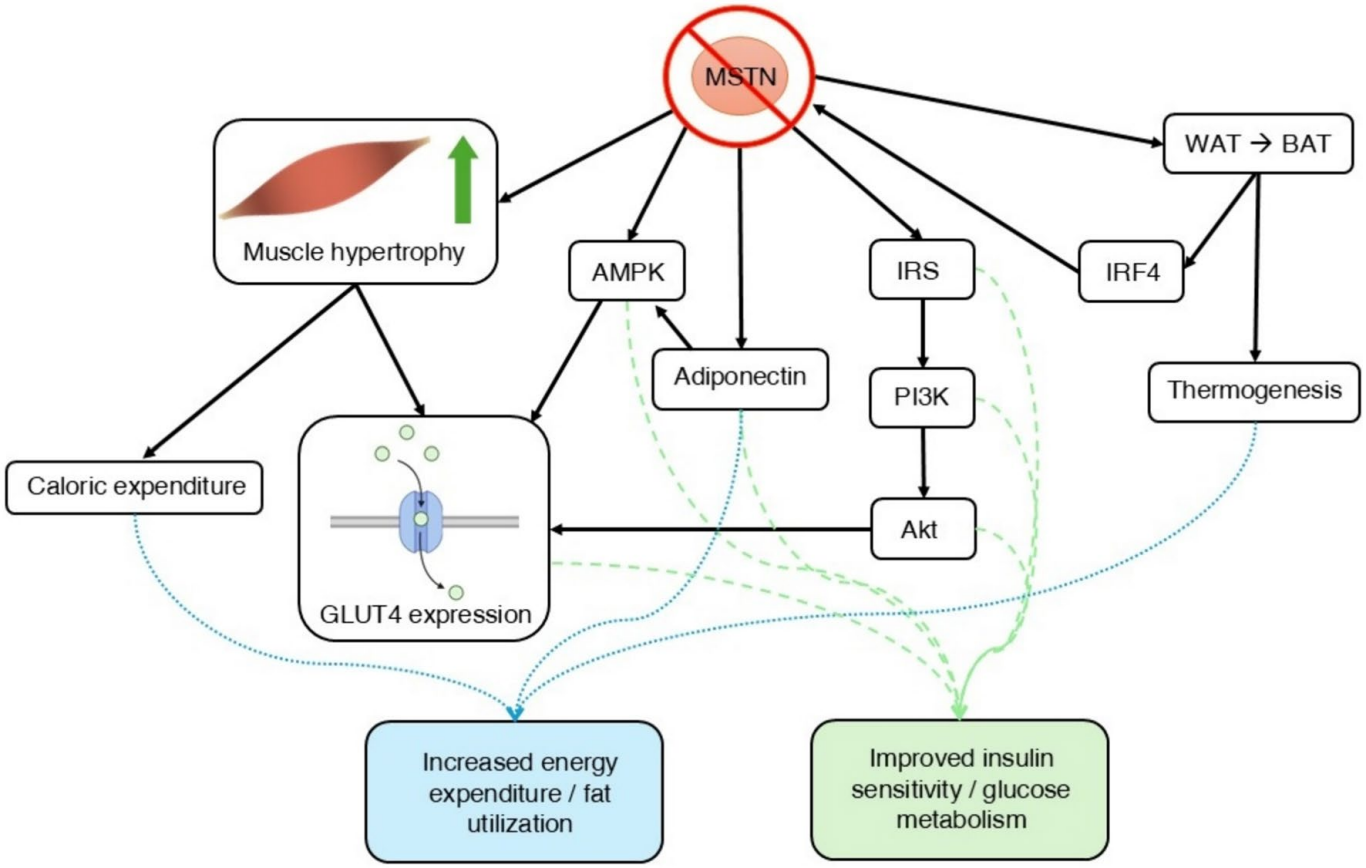
Mechanistic pathways of myostatin inhibition in insulin sensitivity and adipose tissue regulation. Myostatin (MSTN) blockade increases muscle hypertrophy, leading to heightened caloric expenditure and glucose transporter type 4 (GLUT4) expression; it also promotes the activation of adenosine monophosphate-activated protein kinase (AMPK), which facilitates GLUT4 translocation; enhances the expression of adiponectin, further mediating AMPK activation; and leads to the expression of insulin receptor substrates (IRS), activating the phosphoinositide 3-kinase (PI3K)/protein kinase B (Akt) pathway. Akt enhances GLUT4 expression by increasing its translocation to the cell membrane and inhibiting the forkhead box O1 (FOXO1) transcription factor, which in turn increases the transcription of GLUT4. Additionally, MSTN blockade induces brown adipose tissue (BAT) formation in white adipose tissue (WAT) via the expression of BAT markers and thermogenic genes; interferon regulatory factor 4 (IRF4), expressed in BAT, inhibits myostatin expression, and BAT promotes thermogenesis. Increased caloric expenditure, adiponectin, and BAT thermogenesis lead to increased energy expenditure and fat utilization, while GLUT4 expression, AMPK activation, IRS, PI3K, and Akt contribute to improved insulin sensitivity and glucose metabolism; these effects collectively result in an improvement in diabetes and obesity symptoms

Inhibition of MSTN is beneficial for metabolic syndrome and diabetes in various ways. Loss of MSTN function has long been known to reduce total body fat mass. In obesity and T2DM mouse models, loss of MSTN mitigates fat accumulation and deviation from healthy glucose metabolism [119]. Local inhibition by AAV delivery of MSTN propeptide increased glucose uptake and skeletal muscle mass in obese, high-fat-fed mice [120]. Transgenic mouse models with overexpression of MSTN propeptide also resist increases in adipose tissue mass and insulin resistance, which are associated with a high-fat diet, while wild-type counterparts develop prediabetes [121]. Furthermore, crossbreeds between MSTN-knockout and AKITA mice (a model for T1DM) exhibit significantly higher glucose uptake capacity, lower resting blood glucose levels, and reduced diabetic symptoms compared to normal AKITA mice [122]. Additionally, increasing functional muscle mass (assuming it is possible) would be beneficial for treating diabetes, as a loss of skeletal muscle mass is frequently associated with both T1DM and T2DM due to catabolism caused by malfunction of insulin-related issues [123]. MSTN inhibition for the treatment of metabolic disorders in humans has already shown moderate success in clinical trials with bimagrumab. Dosing of bimagrumab in obese adults shows significant increases in lean body mass and reductions in body fat [124, 125]. In obese adults with type 2 diabetes, bimagrumab treatment every four weeks for 48 weeks resulted in a ~ 20% decrease in fat mass, ~ 4% increase in muscle mass, ~ 6.5% decrease in body weight, and ~ 0.8 percentage point decrease in HbA_1c_ level [126]. Ongoing clinical trials with bimagrumab aim to explore its potential in treating obesity; however, no MSTN inhibitor has received approval for use in metabolic syndromes. Expanding the scope of clinical trials targeting obesity, diabetes, and metabolic syndrome is critical for determining MSTN inhibition’s effectiveness in treating these disorders (Fig. 2).

Mechanistically, MSTN inhibition interacts with insulin sensitivity and obesity through both skeletal muscle-dependent and independent mediation. The primary phenotypic change accompanying MSTN suppression is an increase in skeletal muscle, which partitions nutrients away from adipose tissue to support energy requirements for muscle growth. Skeletal muscle is the primary site for insulin-mediated glucose uptake via glucose transporter type 4 (GLUT4) protein [127]. Indeed, MSTN-knockout mice exhibit upregulated GLUT1 (insulin-independent) and GLUT4 (insulin-dependent) proteins, leading to increased glucose uptake [122]. Conversely, active MSTN reduces GLUT4 expression and glucose uptake through muscle atrophy, inhibition of various insulin-related pathways, and downregulation of gene expression [128]. MSTN inhibits the phosphorylation of insulin receptor substrate (IRS) proteins, which reduces the activation of phosphoinositide 3-kinase (PI3K) and downstream protein kinase B (Akt) [129]. Akt promotes the translocation of GLUT4-containing vesicles to the plasma membrane of muscle cells in response to insulin [130]. It also phosphorylates and inhibits Forkhead box O1 (FoxO1), which is a transcription factor that represses GLUT4 gene transcription [131]. MSTN inhibition upregulates the PI3K/Akt pathway, leading to an increase expression of GLUT4 [132, 133]. Additionally, MSTN inhibits the activation of adenosine monophosphate-activated protein kinase (AMPK), a crucial regulator of mitochondrial biogenesis and energy metabolism, which also promotes GLUT4 translocation in response to insulin-independent energy stress [129, 134]. Furthermore, MSTN knockout has been reported to upregulate adiponectin, a regulator of adipocyte energy metabolism that improves insulin sensitivity and stimulates AMPK [134–136]. In our study, transgenic mice overexpressing MSTN propeptide exhibited a significant increase in serum adiponectin levels when fed a high-fat diet, while maintaining normal levels of blood insulin, resistin, and leptin [121].

Although MSTN is not highly expressed in adipose tissue, it plays a significant role in mediating adipose tissue function. Metabolically, MSTN-null mice show increased energy expenditure and leptin sensitivity [137]. Inhibition of MSTN upregulates enzymes involved in lipolysis and mitochondrial fatty acid oxidation, increasing fat breakdown in peripheral tissues, and reducing lipid accumulation [138]. Furthermore, MSTN suppression induces the emergence of brown adipose tissue (BAT) within white adipose tissue (WAT), a fat type known for its thermogenic characteristics, abundance of fat-burning mitochondria, and role in maintaining insulin and glucose homeostasis [138, 139]. MSTN has been shown to mediate the expression of BAT markers and thermogenic genes in WAT, including Ucp1, Prdm16, Pgc-1a, Bmp7, Cidea, Cd137, and Tmem26 [134, 140, 141]. Another possible route of MSTN-mediated BAT formation is the skeletal muscle-derived myokine irisin, which facilitates crosstalk between skeletal muscle and adipose tissue to drive thermogenesis and browning and is increased with inhibition of MSTN [142, 143]. Additionally, MSTN is secreted in BAT and acts as an adipokine, reducing local insulin sensitivity [144]. Furthermore, MSTN is involved in tissue crosstalk between BAT and skeletal muscle through transcription factor interferon regulatory factor 4 (IRF4), which regulates adipogenesis by inhibiting MSTN expression [15]. Expression of IRF4 in BAT is strongly correlated with serum MSTN levels, with loss of IRF4 causing obesity, decreased exercise capacity, and increased serum MSTN [15, 145]. These findings indicate a complex interplay between MSTN and adipose tissue, highlighting distinct effects beyond those mediated by skeletal muscle. Further research is required to fully elucidate the interactions between MSTN inhibition and metabolic disorders.

### Myostatin inhibition in orthopedic disorders

MSTN inhibition may also hold therapeutic benefits for orthopedic diseases. Current literature suggests that MSTN acts as a mediator between muscle and bone metabolism, influencing bone formation and remodeling through paracrine and endocrine mechanisms [8]. MSTN negatively impacts bone formation by inhibiting osteogenic differentiation of mesenchymal stem cells and osteoblasts [146, 147]. It also suppresses chondrogenesis, delaying the transition from cartilage to bone during fracture healing, thereby affecting callus formation and bone regeneration [148]. MSTN is shown to be a positive regulator of osteoclast differentiation, which is responsible for the resorption of aged bone and plays a role in bone degradation in arthritis and osteoporosis [149]. Inhibiting MSTN may have therapeutic applications in promoting bone regeneration and healing in bone fractures, osteoporosis, rheumatoid arthritis, and osteoarthritis. In mice, administration of recombinant MSTN propeptide improved fracture healing in a fibula osteotomy model [150]. Additionally, treatment of young mice with ActRIIB-Fc led to increased bone mass [151]. In a mouse model for rheumatoid arthritis, MSTN is highly expressed in synovial tissues, and transgenic or antibody inhibition of MSTN ameliorates joint destruction and arthritis severity [149]. Although MSTN inhibition has shown promise in animal models for increasing bone mass and improving bone strength, no clinical trials targeting orthopedic diseases have been conducted.

## Summary and future perspectives

Current attempts at clinical application of MSTN inhibitors have encountered challenges with drug design and disease applications. While preclinical studies indicate that inhibiting MSTN can treat muscle wasting diseases, transitioning into human trials remains challenging due to the differences in MSTN concentration between animals and humans, and the necessity of neural input to improve skeletal muscle functionality. Muscular dystrophies, the primary focus of MSTN-inhibition therapeutics to date, stem from genetic-based pathways that may not be easily remedied solely by inhibiting MSTN or augmenting skeletal muscle mass. Future applications of MSTN inhibition must consider the limitations of targeting skeletal muscle mass and explore more suitable disease applications. Obesity, diabetes, metabolic syndromes, and orthopedic diseases are all potential targets for MSTN inhibitors because they do not face the same challenges with serum MSTN concentration and muscle contraction functions, and their benefits are not solely dependent on boosting muscle function. However, these pathological conditions remain understudied in clinical trials. There may still be potential for MSTN inhibitors in managing muscular dystrophy through combination with exercise, dystrophin, or other gene replacement therapies [95, 152–154], or in less severe forms of the condition where elevated levels of residual MSTN are observed, like myotonic dystrophy [105]. These approaches could mitigate the challenges posed by the low MSTN levels in patients with severe muscular dystrophy, which may otherwise reduce the effectiveness of MSTN inhibitors. There are also future application possibilities in non-diseased patients, like targeting MSTN to protect against muscle and bone mass loss during space flight [155]. Regardless of application, future research on MSTN inhibitors should prioritize the development of specific inhibitor designs to mitigate side effects caused by cross-reactivity. Targeting the latent or pro-form of MSTN could offer superior efficacy and reduced cross-reactivity. Most inhibitors tested to date have targeted the mature form or employed broadly reactive receptor-based approaches. Numerous inhibition methods with the potential for greater effectiveness remain unexplored in clinical settings. For instance, MSTN propeptide has demonstrated efficacy as a specific inhibitor but has yet to receive clinical attention. Although MSTN inhibition has yet to fully realize its promise as a muscle-enhancing drug, there is still ample potential for refinement in its therapeutic applications and drug designs.

## Data Availability

No datasets were generated or analysed during the current study.
